# Systematic Understanding of Anti-Aging Effect of Coenzyme Q10 on Oocyte Through a Network Pharmacology Approach

**DOI:** 10.3389/fendo.2022.813772

**Published:** 2022-02-11

**Authors:** Liuqing Yang, Heng Wang, SuJie Song, Hongbin Xu, Yun Chen, Saisai Tian, Yiqun Zhang, Qin Zhang

**Affiliations:** ^1^ Department of Traditional Chinese Medicine (TCM) Gynecology, Hangzhou Hospital of Traditional Chinese Medicine Affiliated to Zhejiang Chinese Medical University, Hangzhou, China; ^2^ Zhejiang Chinese Medical University, Hangzhou, China; ^3^ Second Clinical Medical College, Guangzhou University of Traditional Chinese Medicine, Guangzhou, China; ^4^ Department of Phytochemistry, School of Pharmacy, The Second Military Medical University, Shanghai, China

**Keywords:** oocyte, coenzyme Q10, molecular docking, mechanism, molecular dynamic simulation, aging

## Abstract

**Background:**

Maternal oocyte aging is strongly contributing to age-related decline in female fertility. Coenzyme Q10 (CoQ10) exerts positive effects in improving aging-related deterioration of oocyte quality, but the exact mechanism is unclear.

**Objective:**

To reveal the system-level mechanism of CoQ10’s anti-aging effect on oocytes based on network pharmacology.

**Methods:**

This study adopted a systems network pharmacology approach, including target identification, data integration, network and module construction, bioinformatics analysis, molecular docking, and molecular dynamics simulation.

**Result:**

A total of 27 potential therapeutic targets were screened out. Seven hub targets (PPARA, CAT, MAPK14, SQSTM1, HMOX1, GRB2, and GSR) were identified. Functional and pathway enrichment analysis indicated that these 27 putative targets exerted therapeutic effects on oocyte aging by regulating signaling pathways (e.g., PPAR, TNF, apoptosis, necroptosisn, prolactin, and MAPK signaling pathway), and are involved oxidation-reduction process, mitochondrion, enzyme binding, reactive oxygen species metabolic process, ATP binding, among others. In addition, five densely linked functional modules revealed the potential mechanisms of CoQ10 in improving aging-related deterioration of oocyte quality are closely related to antioxidant, mitochondrial function enhancement, autophagy, anti-apoptosis, and immune and endocrine system regulation. The molecular docking study reveals that seven hub targets have a good binding affinity towards CoQ10, and molecular dynamics simulation confirms the stability of the interaction between the hub targets and the CoQ10 ligand.

**Conclusion:**

This network pharmacology study revealed the multiple mechanisms involved in the anti-aging effect of CoQ10 on oocytes. The molecular docking and molecular dynamics simulation provide evidence that CoQ10 may act on these hub targets to fight against oocytes aging.

## Introduction

Aging leads to a gradual and continuous decline of physiological processes. Oogenesis is one of the first processes to fail during human aging. Human oocyte development begins in the early fetal life, reaches its peak at 20 weeks of gestation, and remains dormant before complete meiosis I (1). Over time, the quality and quantity of these oocytes gradually declined, concurrently with the reduced reproductive ability and increased occurrence of aneuploidy, miscarriage, and birth defects (2–4). In contrast, the aging-related decline in fecundity was negated in patients using oocytes obtained from healthy young donors (5). These data suggest that the age-related decline in fertility is strongly attributed to a decline in the oocyte quality.

Oocytes accumulate a large pool of maternal RNA and protein during meiotic maturation to support early embryogenesis before embryonic genome activation (6, 7). Therefore, oocyte quality is a critical prerequisite for fertilization and subsequent embryo development (8). In the clinic, the paradigm that aged oocytes represent low‐quality oocytes is widely confirmed. Data from a reproductive center in China shows that among 20,687 women receiving IVF, the complete full cycle live birth rate of women under 31 years old was 63.81%, while the rate of women over 40 years old decreased to 4.71% (3). However, due to education, social, economic, and other factors, the average age of first-time mothers is rising worldwide, which poses a great challenge to reproductive medicine (9, 10).

Various interventions have been proposed to improve oocyte quality, thereby improving pregnancy outcomes in oocyte aging-related infertility. As the third most consumed dietary supplement, coenzyme Q10 (CoQ10) has attracted global interest due to its crucial role in mitochondrial bioenergy, anti-oxidation, anti-aging, and immune system regulation. In the meantime, the role of CoQ10 in human reproduction has received widespread attention, especially in improving oocyte quality and counteracting oocyte aging (11–14). Turi et al. confirmed for the first time that CoQ10 is present in human follicular fluid, and its level was significantly higher in the mature oocytes versus dysmorphic oocytes; correspondingly, the CoQ10 level in grade I-II embryos was significantly higher than that of III-IV embryos (15). A further clinical study conducted by Giannubilo et al. indicated that oral supplements with CoQ10 increased the content of CoQ10 in follicular fluid, and CoQ10 in follicular fluid was positively correlated with oocytes quality (12). In addition, pretreatment with CoQ10 significantly increased the number of retrieved oocytes, fertilization rate, and the number of high-quality embryos in women with poor ovarian response (POR) during the IVF-ICSI cycle (13). Rodent studies have also confirmed that CoQ10 counteracts physiological ovarian aging and improves ovarian reserve through multiple mechanisms (16–19).

**Figure 1:**
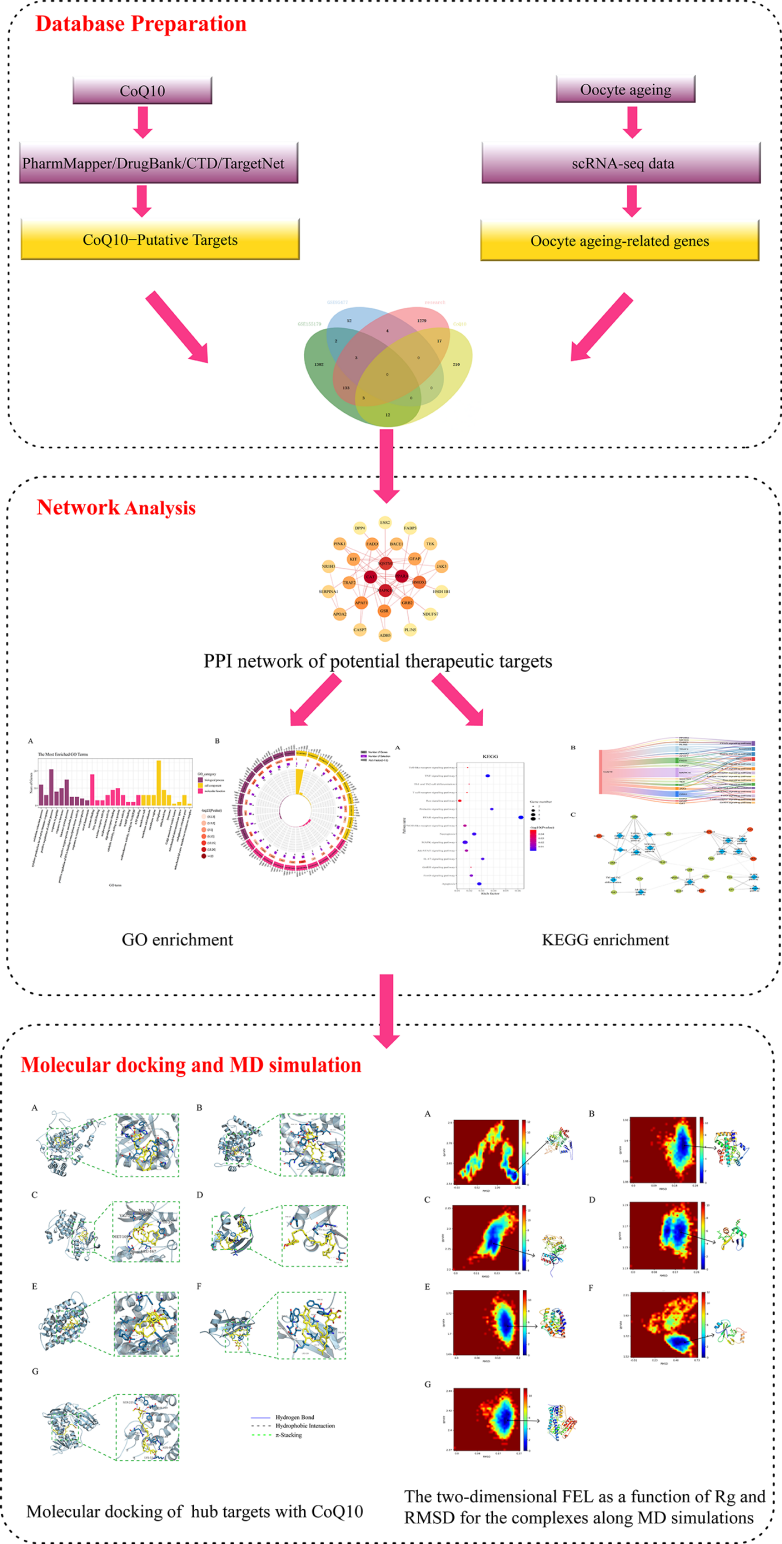
Workflow diagram of this research.

Currently, although anti-oocyte aging activities exerted by CoQ10 have been reported, details of biomarkers and the biological pathways through which CoQ10 counteracts oocyte aging are limited. In previous studies, a network pharmacology-based approach was successfully used to reveal all candidate targets, functions, and potential therapeutic mechanisms of drugs (20–22). In the present study, system network pharmacology combined with molecular docking was performed to reveal the potential targets and therapeutic mechanisms of CoQ10 against oocyte aging. The workflow is shown in **Figure 1**.

## Materials and Methods

### Selection of Oocyte Aging-Associated Genes from scRNA-Seq Results

Oocyte aging-associated genes were searched in the GEO database (https://www.ncbi.nlm.nih.gov/geo/), and the series (GSE96477 and GSE155179) were selected. The GSE95477 and GSE155179 include single-cell RNA sequencing (scRNA-seq) data of MII human oocytes from younger (≤30 years) and older (≥40 years) patients. DESeq2 software (version 1.32.0) was used to determine differentially-expressed genes (DEGs) in all datasets. Genes with a *P* value <0.05 and |log 2 (fold change) | >1 were considered to be DEGs, namely, oocyte aging-related genes.

In addition, we searched Pubmed databases (https://pubmed.ncbi.nlm.nih.gov) using human oocyte aging and scRNA-seq as keywords. We include Zhang’s research which isolated mRNA from human oocytes obtained from old and young patients for scRNA-seq and subsequently identified differentially expressed genes (23). In summary, three scRNA-seq data sets were included for differential gene analysis. All differentially expressed genes were integrated into oocyte aging genes.

### Identification of Putative CoQ10 Targets

Simplified molecular-input line-entry specification (SMILES) information and 3D structure of CoQ10 were obtained from PubChem database (https://pubchem.ncbi.nlm.nih.gov/). PharmMapper server (https://lilabecust.cn/pharmmapper/), Drug bank (https://go.drugbank.com/),TargetNet (http://targetnet.scbdd.com/), and Comparative Toxicogenomics Database (CTD, https://ctdbase.org/) were used to identify possible targets for CoQ10.

### Construction of Protein-Protein Interaction Network

The oocyte aging-rated genes and the CoQ10 action targets were intersected to obtain the genes of CoQ10 against oocyte aging. The Venn diagram was drawn by using the bioinformatics platform Jvenn (http://jvenn.toulouse.inra.fr/app/index.html) and the protein-protein interaction (PPI) network of the common targets was obtained by using the STRING database (https://string-db.org/). The species was limited to Homo sapiens, and we selected the combined score >0.4. The Cytoscape 3.8.2 was used to construct the PPI network.

### GO and KEGG Pathway Enrichment

The OmicShare tools (https://www.omicshare.com/), a free data analysis platform, were used to analyze the GO and KEGG enrichment of the potential therapeutic targets. The Go analysis includes biological processes (BP), cellular components (CC), molecular function (MF).

### Construction of Network

Two networks were constructed: (1) the PPI network was constructed using the genes of CoQ10 against oocyte aging; (2) the CoQ10-targets-pathways network, this network was divided into different functional modules using the Community Cluster (Glay) algorithm of clusterMaker2.

### Molecular Docking of the Active Components-Targets

The 3D structures of protein receptors were obtained from the RCSB Protein Data Bank (https://www.rcsb.org/) (24). PyMol software (v2.4.0) was applied to remove the heteroatom and water molecules from the proteins. The 2D structure of CoQ10, used as a ligand, was downloaded from the PubChem website (https://pubchem.ncbi.nlm.nih.gov/) (25). AutoDockTools (v1.5.7) software was used to convert the above structure into a pdbqt file. The prediction of binding sites were obtained from Deepsite website (https://www.playmolecule.org/deepsite/). Molecular docking was completed by Autodock vina software (v1.1.2) (26). The complexes were then observed and plotted using PyMol software.

### Molecular Dynamic Simulation

The conformations with the highest scores obtained from the molecular docking (protein-ligand complexes) were further subjected to molecular dynamic (MD) simulations using Gromacs 2020.6. (27). The CHARMM36 force field was used to generate the parameters of the protein (28). The Acpype Server (https://www.bio2byte.be/acpype/) (29) was used for ligand topology. The protein-ligand complex system was built in a dodecahedron box and filled with three-point water model. Sodium ions or chloride ions neutralize the charge of the system, making it electrically neutral. The system was energy-minimized for 50,000 steps at the temperature of 300k and equilibrated by 100 ps of NVT and NPT simulations subsequently. Then, a production run of 30 ns was performed with a step length of 2.0 fs, and the coordinate track file was saved every 10 ps. The root-mean-square deviation (RMSD) and root-mean-square fluctuation (RMSF) throughout the trajectory were analyzed using the gmx rms and rmsf, respectively. The g_mmpbsa program was used to evaluated the binding free energy of the complexes.

## Results

### Oocyte Aging-Related Genes

The GSE95477 data set derived from the GEO database was analyzed and 61 DEGs were obtained according to the cut-off criteria. 1455 DEGs of GSE155179 date are obtained *via* the same method.

In addition, we downloaded the supplementary data of Zhang’s research (23). Then, we re-screened DEGs according to the cut-off criteria and obtained 1439 DEGs. Finally, all the obtained DEGs were merged, 2807 oocyte aging-related genes were identified **(** **Supplementary 1** **).**

### CoQ10−Putative Targets

A total of 242 CoQ10 targets were obtained after removing duplications from the PharmMapper, DrugBank, TargetNet and CTD databases **(** **Supplementary 2** **)**.

### PPI Network of the Potential Therapeutic Targets

**Figure 2:**
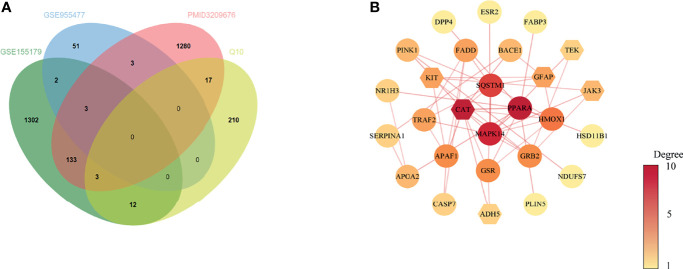
Venn diagram and PPI network of potential therapeutic targets. **(A)** Venn diagram of intersected targets of oocyte aging and CoQ10. **(B)** PPI network of potential therapeutic targets. The nodes’ colors are illustrated from yellow to red in descending order of degree values.

Based on the results above, 31 common target genes of CoQ10-putative targets and oocyte aging-related genes were identified after removing EDN1which was up-regulated in GSE155179 but down-regulated in Zhang’s research **(** **Figure 2A** **)**.

The PPI network of common target genes consisted of 27 nodes and 51 edges, and 4 free nodes (targets) were removed.

The 27 nodes of the PPI network were identified as potential therapeutic targets **(** **Figure 2B** **)**. The CytoNCA, an app plugin in Cytoscape, was used to analyze the topological parameters of the network **(** **Supplementary 3** **)**.

To find the hub targets in this complex biological network, the topological parameters including degree centrality (DC), betweenness centrality (BC), closeness centrality (CC), eigenvector centrality (EC), local average connectivity-based method (LAC), network centrality (NC) were analyzed. As a result, a total of seven genes (PPARA, CAT, MAPK14, SQSTM1, HMOX1, GRB2, and GSR), were obtained according to the median values of DC, BC, CC, EC, LAC, NC **(** **Supplementary 4** **)**. The seven genes were used for the subsequent molecular docking and molecular dynamic simulation.

### GO Enrichment Analysis

**Figure 3:**
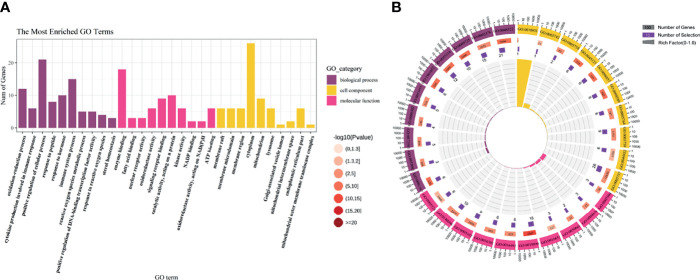
GO enrichment analysis. **(A)** The top 10 significantly enriched terms of each part. BP, biological process, CC, cell component, MF, molecular function. **(B)** The first lap indicates top 10 GO term, and the number of the genes corresponds to the outer lap. The second lap indicates the number of the genes in the genome background and -lg (p-value). The third lap indicates the DEGS. The fourth lap indicates the enrichment factor of each GO term.

To further study the molecular mechanism of CoQ10 against oocyte aging, GO enrichment of the potential therapeutic targets was performed based on BP, CC, and MF (**Figure 3A** **)**. In terms of BP, the anti-aging effect of CoQ10 on oocyte mainly involves the regulation of reactive oxygen species, participation of immune process, and response to hormone etc. The MF mainly involves nuclear transcription, cytokine activity, and ATP binding etc. The CC is mainly manifested in cell membrane, cytoplasm, and mitochondrion etc. (**Figure 3B**).

### KEGG Enrichment Analysis

**Figure 4:**
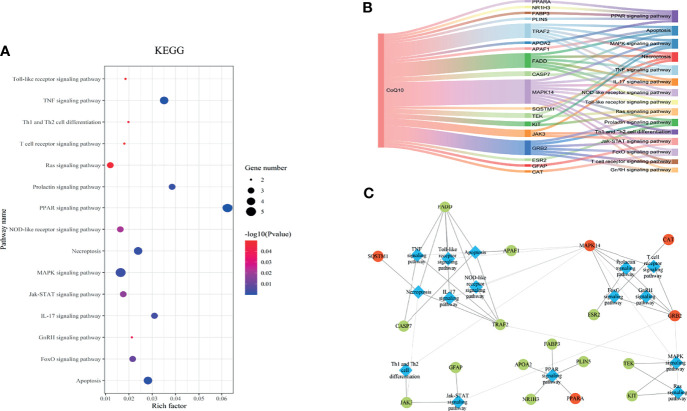
The KEGG pathway analysis of the 27 potential therapeutic targets. **(A)** The 15 significant pathways. The bubbles’ sizes are indicated from big to small in descending order of the count of the potential targets enriched in the pathways. The bubbles’ colors are indicated from red to blue in descending order of -lg (*p-*value). **(B)** CoQ10-targets-pathways network. The width of the line is proportional to the number of connected points. **(C)** Module analysis of the target-pathway network. The diamond nodes represent the pathways, and the ellipse nodes represent the targets. The red nodes represent the hub genes obtained from the PPI network of potential therapeutic targets.

The KEGG pathway enrichment was identified based on the potential therapeutic targets. 53 pathways with *P* value < 0.05 were obtained. After data screening, 10 significant pathways were selected **(** **Figure 4A** and **Table 1** **)**.

**Table 1:** The KEGG results.

Pathway class	Pathway	p-value
Cell growth and death	Apoptosis	0.0010556
Cell growth and death	Necroptosis	0.001923
Endocrine system	PPAR signaling pathway	4.98E-06
Endocrine system	Prolactin signaling pathway	0.001977
Endocrine system	GnRH signaling pathway	0.036979
Immune system	IL-17 signaling pathway	0.003678
Immune system	NOD-like receptor signaling pathway	0.02113
Immune system	Th1 and Th2 cell differentiation	0.042137
Immune system	Toll-like receptor signaling pathway	0.04755
Immune system	T cell receptor signaling pathway	0.049945
Signal transduction	TNF signaling pathway	0.000462
Signal transduction	MAPK signaling pathway	0.002634
Signal transduction	FoxO signaling pathway	0.009981
Signal transduction	Jak-STAT signaling pathway	0.017418
Signal transduction	Ras signaling pathway	0.046217

Then, the CoQ10-targets-pathways network was constructed after sorting out the genes enriched in each pathway **(** **Figure 4B** **)**. To further investigate the mechanism of CoQ10 against oocyte aging, five functional modules were constructed using the Glay algorithm of clusterMaker2 in the targets-pathways network **(** **Figure 4C** **)**. Modules 1 consisted of six pathways, including the TNF signaling pathway (has04668), apoptosis (has04210), necroptosis (has04217), IL-17 signaling pathway (has04657), Toll-like receptor signaling pathway (has04620), and NOD-like receptor signaling pathway (has04621). Modules 2 comprised 4 pathway, including the prolactin signaling pathway (has04917), T cell receptor signaling pathway (has04660), FaxO signaling pathway (has04068), and GnRH signaling pathway (has04912). Module 3 consisted of 2 pathways, including the Th1 and Th2 cell differential (has04658) and Jak-STAT signaling pathway (has04630). Module 4 comprised PPAR signaling pathway (has03320). Module 5 comprised 2 pathways, including the MAPK signaling pathway (hsa04010) and Ras signaling pathway (hsa04014).

### Molecular Docking

**Figure 5:**
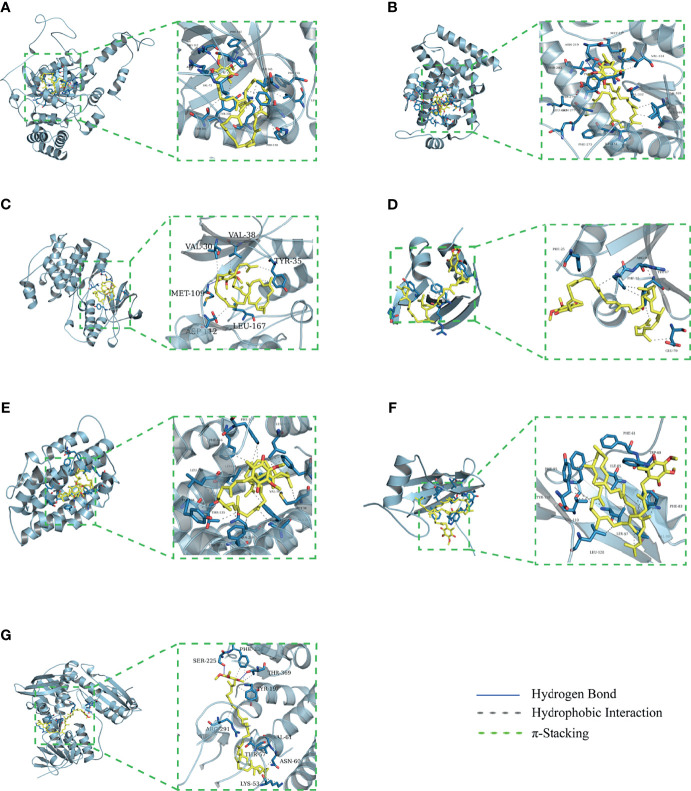
Molecular docking of 7 hub targets with CoQ10. **(A)** The binding poses of CAT complexed with CoQ10. **(B)** The binding poses of PPARA complexed with CoQ10. **(C)** The binding poses of MAPK14 complexed with CoQ10. **(D)** The binding poses of SQSTM1 complexed with CoQ10. **(E)** The binding poses of HMOX1 complexed with CoQ10. **(F)** The binding poses of GRB2 complexed with CoQ10. **(G)** The binding poses of GSR complexed with CoQ10.

The binding energy can be calculated to predict their affinity. Generally, the lower the binding energy of the ligand and the receptor, the more stable the binding conformation. As presented in **Table 2**, all binding affinities are lower than -5 kcal/mol, indicating that CoQ10 has a strong binding activity to the seven hub genes, and the binding conformation is stable. **Figure 5** shows the binding mode of CoQ10 with the hub targets.

**Table 2:** Molecular docking parameters and binding free energy.

Targets	PDB ID	Box center (x, y, z)/Å	Affinity/(kcal/mol)
CAT	1DGF	24.9, 57.6, 56.2	−9.4
PPARA	6KXX	13.6, −11.6, −28.8	−5.1
MAPK14	5ETI	0.5, 12.339, −33.37	−5.5
SQSTM1	6JM4	−31.519, −15.357, 25.928	−5.3
HMOX1	3CZY	5.8, −21.6, 2.7	−8.7
GRB2	2H46	14.9, 55.5, 36.0	−7.4
GSR	3DK9	14.4, 15.8, 20.46	−8.4

### MD Simulation

To further validate these molecular docking simulation results, MD simulations were performed. The RMSD and RMSF of the backbone were used to scrutinize the stability of the structure model of the CoQ10 and its targets complex. The trajectory files in MD simulation were made as movies (**Supplementary 5** **)**.

**Figure 6:**
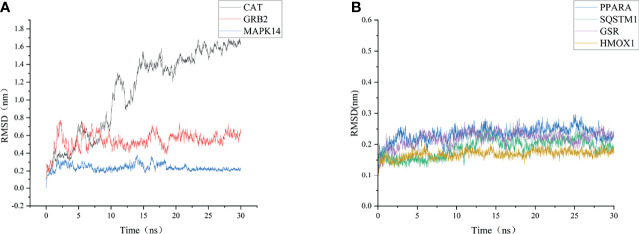
RMSD change of targets backbone atoms in MD simulation. **(A)** RMSD of CAT-CoQ10 complex, RMSD of GRB2-CoQ10 complex, RMSD of MAPK14-CoQ10 complex **(B)** RMSD of HMOX1-CoQ10 complex, RMSD of PPARA-CoQ10 complex, RMSD of SQSTM1-CoQ10 complex, RMSD of GSR-CoQ10 complex.

**Figure 7:**
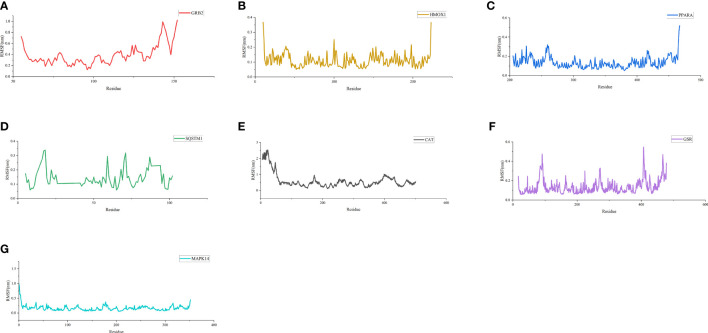
RMSF of residues. **(A)** RMSF of GRB2-CoQ10 complex. **(B)** RMSF of HMOX1-CoQ10 complex. **(C)** RMSF of PPARA-CoQ10 complex. **(D)** RMSF of SQSTM1-CoQ10 complex. **(E)** RMSF of CAT-CoQ10 complex. **(F)** RMSF of GSR-CoQ10 complex. **(G)** RMSF of MAPK14-CoQ10 complex.

As shown in **Figure 6**, the RMSD value fluctuates within 0.2 nm after 20 ns, and the system gradually reaches equilibrium. In addition, the average fluctuations of the protein residues were evaluated as the RMSF during the MD simulation **(** **Figure 7** **)**.

According to the RMSD values, we extracted the 20-ns to 30-ns trajectory for analysis. Van Der Waals interaction, electronic energy, polar solvation energy, solvent-accessible, and surface area (SASA) energy between CoQ10 and targets were calculated using g-MMPB/SA software. The results were shown in **Table 3**. It is observed that the van der Waals interaction, electronic energy, and SASA energy promote the binding of CoQ10 to the target protein, followed by electronic energy and SASA energy, while the polar solvation energy have the opposite effect.

**Table 3:** Binding free energy of CoQ10 and targets (kJ/mol).

Target	Van der Waal energy	Electrostatic energy	Polar solvation energy	SASA energy	Binding energy
CAT	−393.656 ( ± 16.389)	−29.921 ( ± 17.322)	233.195 ( ± 23.928)	−42.862 ( ± 2.053)	−233.244 ( ± 21.748)
PPARA	−446.740 ( ± 23.404)	−20.106 ( ± 8.231)	168.004 ( ± 14.808)	−48.462 ( ± 1.796)	−347.305 ( ± 23.618)
MAPK14	−293.871 ( ± 13.687)	−18.961 ( ± 4.339)	164.676 ( ± 10.668)	−30.949 ( ± 1.073)	−179.105 ( ± 15.678)
SQSTM1	−265.856 ( ± 17.818)	−7.122 ( ± 13.431)	182.525 ( ± 47.290)	−30.460 ( ± 2.025)	−120.913 ( ± 43.240)
HMOX1	−358.136 ( ± 22.253)	−15.838 ( ± 12.736)	186.772 ( ± 34.374)	−41.255 ( ± 1.841)	−228.458 ( ± 37.123)
GRB2	−241.451 ( ± 18.234)	−5.154 ( ± 11.358)	100.810 ( ± 22.315)	−29.303 ( ± 2.330)	−175.098 ( ± 17.198)
GSR	−372.167 ( ± 21.208)	−6.454 ( ± 7.347)	310.453 ( ± 31.425)	−46.396 ( ± 1.890)	−114.564 ( ± 24.918)

**Figure 8:**
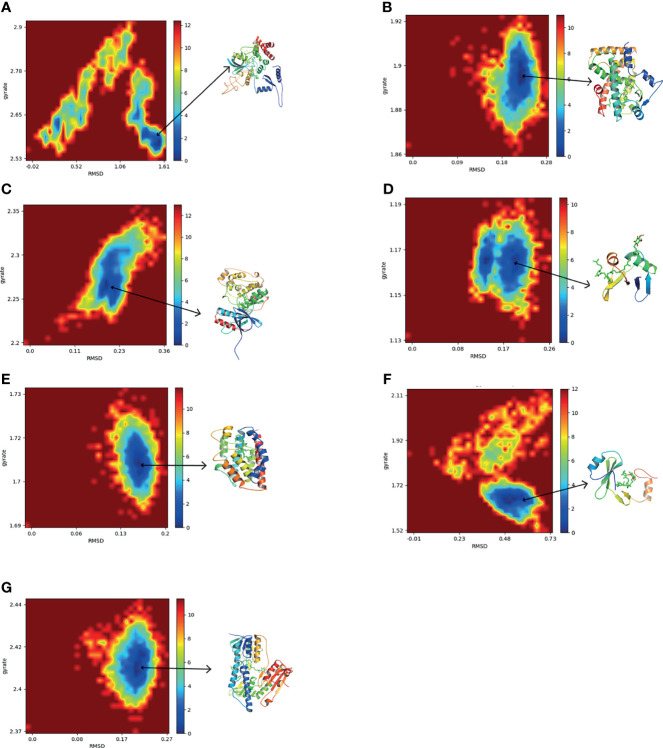
The two-dimensional FEL as a function of Rg and RMSD (defined in the text) for the complexes along 30 ns MD simulations. Snapshots from minimum energy wells were extracted. **(A)** CAT-CoQ10 complex. **(B)** PPARA-CoQ10 complex. **(C)** MAPK14-CoQ10 complex. **(D)** SQSTM1-CoQ10 complex. **(E)** HMOX1-CoQ10 complex. **(F)** GRB2-CoQ10 complex. **(G)** GSR-CoQ10 complex.

To further explore the conformation changes of CoQ10 and its targets complex, we constructed the free energy landscapes of the complex and defined two reaction coordinates of the free landscape map: one is RMSD, which shows the stability of the protein structure; the other is the radius of gyrate (Rg), which reflects whether the complex is folded stably. The energy landscape was calculated along these two reaction coordinates using the gmx sham tools **(** **Figure 8** **)**.

As shown in **Figure 8**, the blue area indicates the minimum value of free energy, while the cyan and green areas indicate the metastable conformational state. For all complexes, a higher blue color is observed, which represents that these complexes were stable.

The MD simulation study further evaluated the stability of the interaction between the hub targets and the CoQ10 ligand predicted by the docking experiment. In addition, free energy calculation provides an assessment of the binding affinity of hub targets to CoQ10. In our study, MD simulations showed that the structures of all complexes are very stable. Among them, the PPARA-CoQ10 complex has the lowest binding free energy, indicating that its binding affinity is the highest and may play a major role in CoQ10’s anti-aging effect on oocytes.

## Discussion

The reproductive potential in women gradually declines but significantly beginning approximately in their early 30s. However, many young women choose to postpone marriage and/or childbirth driven by social trends. Aging-related deterioration of oocyte quality is one of the great challenges in reproductive medicine. As mentioned previously, increasing evidence has shown that CoQ10 effectively improve oocyte quality and counteract oocyte aging, but the mechanisms underlying these benefits are still not fully understood. In recent years, the emergence of scRNA-seq technology has dramatically expanded our knowledge of normal and disease-related physiological processes (30, 31). Therefore, in the present study, for the first time, we integrated scRNA-seq data from younger (≤30 years), and older (≥40 years) female oocytes to obtain oocyte aging-related genes. Next, a comprehensive network pharmacology method was utilized to elucidate the mechanism of action of CoQ10 against oocyte aging. According to the network pharmacology results, PPARA, CAT,MAPK14,SQSTM1,HMOX1,GRB2, and GSR play vital roles in protecting against oocyte aging *via* CoQ10. In addition, the molecular docking of the hub genes and CoQ10 exhibited high affinities. Moreover, MD simulations showed that the structures of all complexes (seven hub genes and CoQ10 complexes) are very stable, implying that the seven hub genes may be highly correlated in against oocyte aging with CoQ10.

### The Hub Targets of CoQ10’s in Countering Oocyte Aging

PPARA (also known as PPARα) and two additional PPAR isotypes, PPARβ and PPARγ, are a family of nuclear hormone receptors belonging to the steroid receptor superfamily. PPARα is the first PPAR identified by Issemann and Green in 1990 (32). Since then, it was learned that all the three PPAR isotypes are expressed in the hypothalamic-pituitary-gonadal axis and play a critical role in regulating the proliferation of the different ovarian cells, gametogenesis, steroidogenesis, ovulation, and corpus luteum regression (33, 34). For example, a study using PPARα (−/−) knockout mice has shown that PPARα-dependent pathways play a key role in mediating DEHP’s regulation of ovarian ESR1 expression (35). In addition, the pan-agonist of PPARα, bezafibrate, prevents porcine oocytes (in *in vitro*-matured) aging through its antioxidant properties and mitochondrial protection (25). Tiefenbach, et al. found that CoQ10 and its derivatives serve as direct partial agonists for both PPARγ and PPARα (36). Consistently, our molecular docking results showed that PPARα has a good binding ability with CoQ10. Further MD simulations showed that the PPARA-CoQ10 complex has the lowest binding free energy, indicating that its binding affinity is the highest, and may play a major role in CoQ10’s anti-aging effect on oocytes.

CAT, which encodes catalase, is a critical endogenous antioxidant enzyme defence against oxidative stress (37, 38). Oxidative stress is caused by the imbalance between the production and destruction of reactive oxygen species (ROS), directly damages the cellular proteins, lipids, and DNA, and thus accelerates oocyte apoptosis and deteriorates oocyte quality (39–42). CoQ10 is one of the representative antioxidants. Several studies have shown that CoQ10 treatment significantly increased the CAT activity, improved preantral follicles viability, and protected ovarian reserve by inhibiting oxidative stress (43–45).

MAPK14 (also known as p38MAPKα) is a member of the MAP kinase family. It is known that activated MAPK can regulate the expression of gonadotropin synthesis genes LHβ and FSHβ, and induce follicle growth and ovulation (67, 68).However, numerous studies have shown that specific MAPK14 inhibitors could block FSH-induced MAPK 3/1 phosphorylation in granulosa cells (GCs). Consistently, the MAPK14 inhibitor SB203580, blocks cumulus cell-oocyte (COC) expansion and inhibits the induction of genes (FSH, AREG, and PGE2) critical for this process in a dose-dependent manner (46). In summary, MAPK14 plays an essential role in COC expansion, oocyte maturation, and steroidogenesis.

Autophagy is a ubiquitous dysfunctional cell components degradation process that plays a critical role in cellular homeostasis (47). SQSTM1, also known as p62, is a classical receptor of autophagy, and its role in regulating oxidative stress and promoting oocyte longevity has attracted widespread attention (48–50). Recent evidence indicates that endogenous SQSTM1/p62 is present as gel-like structures, which serves as a platform for autophagosome formation and anti-oxidative stress response (49). In the experiment of spermidine-induced cytoprotective autophagy of female germline stem cells (FGSCs), Yuan et al. found that spermidine, *via* up-regulating the expression of SQSTM1/p62, ameliorate the cellular senescence of FGSCs caused by OS (51).

GCs have long been known to provide the physical support and microenvironment required for oocyte ​development. Cumulative evidence has shown that HMOX1, commonly HO-1, has important antioxidant and antiapoptotic in human and animal ovarian GCs (52, 53). GRB2 is a critical molecule in intracellular signal transduction that participates in organogenesis, development, and induction of meiosis in oocytes (54, 55). GSR is a key member of the glutathione antioxidant defense system. Studies have shown that the down-regulation of antioxidant genes, including GSR, is a unique aging feature of early-stage oocytes (56, 57).

In summary, CoQ10 may act on these hub targets to fight against oocytes aging as verified by the molecular docking and MD Simulation.

### Important Pathways and Functional Modules of CoQ10’s Putative Targets

The molecular mechanisms of oocyte aging are greatly complicated, involving various biological processes and pathways. The 27 therapeutic targets of CoQ10 against oocyte aging screened in this study mainly participate in the oxidation-reduction process, mitochondrion, enzyme binding, reactive oxygen species metabolic process, ATP binding, electron transfer activity, and membrane raft et al. Notably, of all biological processes, oxidative stress and mitochondrial-related biological functions seem to be the most relevant.

**Figure 9:**
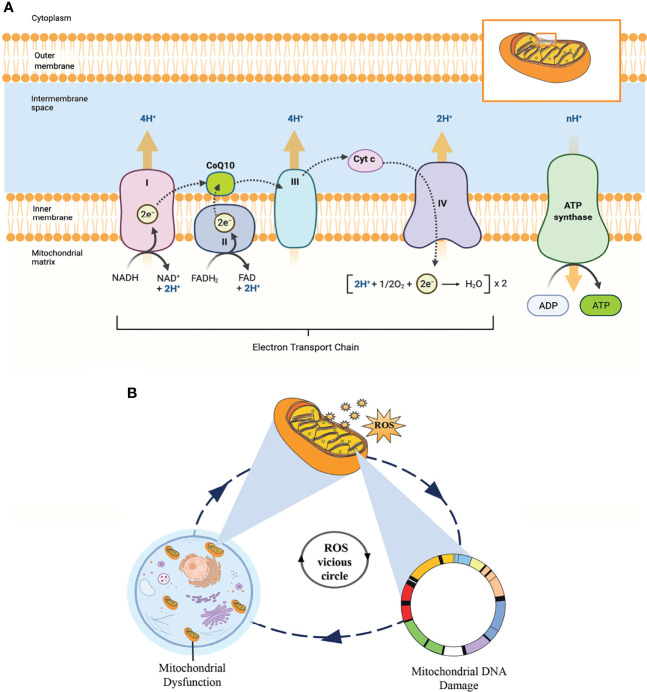
**(A)** The process of oxidative phosphorylation in the mitochondria. This figure shows the central role of CoQ10 as an electron and proton transporter in the mitochondrial respiratory chain. **(B)** This “ROS vicious cycle” depicts the production of mitochondrial ROS causing oxidative damage that leads to mitochondrial DNA damage, mitochondrial dysfunction, and further increasing the production of ROS.

Mitochondria are the most abundant organelles in mammalian oocytes. They act as energy factories in the cells and provide energy for oocyte meiosis maturation, fertilization, and embryonic development through oxidative phosphorylation (OXPHOS). In addition, the mitochondrial genome only comes from the mother, mitochondrial DNA (mtDNA) copy number can be used as a potential biomarker to predict oocyte quality and embryo viability (58, 59). Therefore, reproductive specialists proposed that mitochondrial microinjection is the most promising therapy to restore the quality of oocytes and overcome age-related infertility (60). As shown in **Figure 9A**, OXPHOS involves the action of the mitochondrial electron transport chain (ETC), which consists of five protein complexes located on the inner mitochondrial membrane. As an essential component of the ETC and ATP synthesis, CoQ10 acts as an electron and proton transporter in the ETC, transferring electrons derived from complexes I and II to complex III. Complex III pumps protons across the membrane and transfers to Cyt c. Then, complex IV transfers the electrons received from Cyt c to oxygen, consuming 2 protons on the matrix side and transferring 2 protons to the membrane space simultaneously, generating an electrochemical proton gradient. At last, ATP synthase uses this proton gradient to form ATP from ADP and phosphate, providing energy for all oocyte events.

According to the free radical theory, oxidative stress caused by elevated intracellular levels of ROS is the most significant contributor to oocyte aging. However, mitochondria are the principal source of cellular ROS, especially through electron leakage from Complexes I and III, and the mitochondria themselves are the primary targets of ROS detrimental effects. In addition, mtDNA is particularly vulnerable to ROS-mediated oxidative damage due to the lack of histone protection. ROS is mainly produced in the matrix side of the inner mitochondrial membrane and overlaps with mtDNA positions. This condition promotes the formation of mtDNA-protein cross-linking mediated by ROS, which is one of the most serious forms of DNA damage. In turn, mtDNA damage directly impairs electron transfer chain and ROS production in the mitochondria, forming a “ROS vicious circle” (**Figure 9B**). In the clinic, age-related increases in ROS have been found in the follicular fluid of women, while the levels of antioxidants (CAT, SOD, and GPX) in ovaries are reduced with female age (61, 62). The imbalance between ROS and antioxidants leads to a decline in oocyte quality, an essential factor affecting oocyte quality. Fortunately, CoQ10, as a lipid-soluble antioxidant, can scavenge ROS and inhibit DNA oxidation.

Interestingly, there is a tendency to CoQ10 in a variety of mammalian tissues to be reduced with age (especially after the age of 30 in humans) (63). This age-related decline in the CoQ10 level seems to coincide with the time when female fertility began to decline, suggesting a contribution of the reduced CoQ10 level to ovarian aging. To date, lots of studies have revealed that CoQ10 supplements may increase ATP content in oocytes, improve oocyte quality, and significantly delays reproductive aging (16).

Furthermore, the KEGG pathway analysis indicated that the PPAR signaling pathway was the most enriched. Other related pathways include the TNF signaling pathway, Apoptosis, Necroptosis, Prolactin signaling pathway, MAPK signaling pathway, etc. Previous studies have shown that the PPAR signaling pathway is involved in activating the most abundant fertility reserve in female mammals-dormant primordial follicles (64). In addition, between the delicate and intricate game of equilibrium of antithetic peroxidation and antioxidation forces, the PPAR signaling pathway stands out as a central player devoted to against oxidative stress (65). Furthermore, the PPAR signaling pathway also plays a key role in mitochondrial function. In the diabetes-induced atrial, the agonist of PPAR-γ significantly increases mitochondrial biogenesis-related transcription factors (PGC1α, NRF1–2, TFAM) and mtDNA copy number and reduces mitochondrial ROS production (66).

To further understand the CoQ10 mechanisms in improving oocyte quality, five densely linked functional modules were identified based on the target pathway network, as shown in **Figure 6C**. These modules may be particularly relevant for CoQ10 against oocyte aging, as proteins in a given module may collectively participate in specific biological functions. The first module consists of pathways in the immune system, cell growth and death, and related signaling pathways. The second module consists of pathways in the endocrine and immune system. The third module includes pathways in signal transduction and the immune system, and the fourth mainly involves the PPAR signal pathway.

The last module includes pathways in the signal transduction related to GCs proliferation and oocyte meiosis. Among them, three-fifths of the modules involve the immune system. Specifically, immune system-associated cells and molecules exist in the hypothalamus-pituitary–ovarian axis, regulating the development of fetal and adult primordial follicles. Moreover, Bukovsky et al. demonstrated that immune cells and their secreting factors are an important part of the ovarian germline stem cells (OSC) niche. They participate in the asymmetric division of OSCs and promote the division, proliferation and migration of germ cells and the growth of follicles (67, 68). These modules reflected CoQ10’s effects on endocrine and immune regulation, anti-apoptosis, and GCs proliferation and oocyte meiosis.

## Conclusions

**Figure 10:**
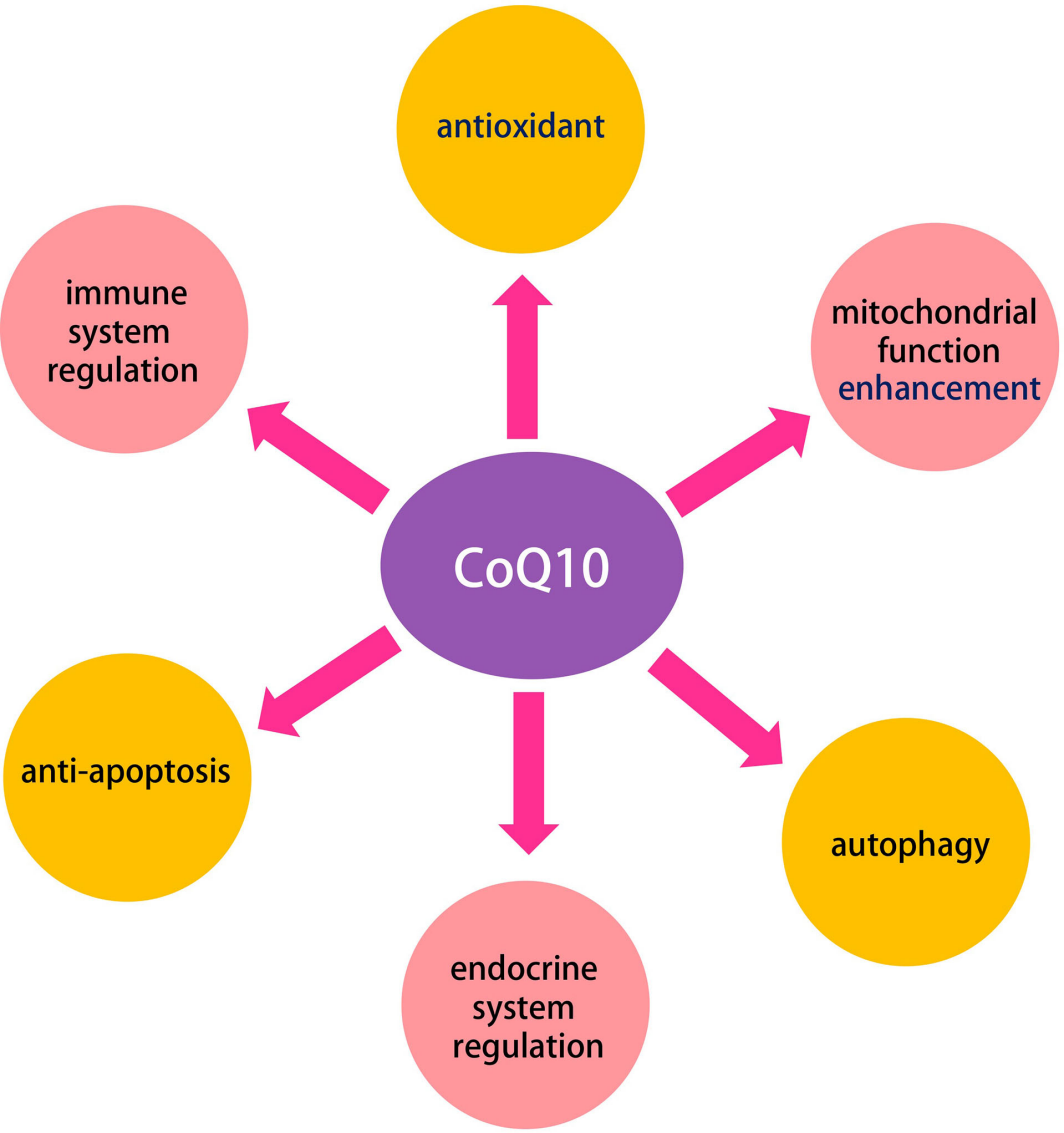
The multiple roles of CoQ10 in oocyte.

In the present work, we found that the potential mechanisms of CoQ10 in improving aging-related deterioration of oocyte quality are closely related to antioxidant, mitochondrial function enhancement, autophagy, endocrine and immune system regulation, and anti-apoptosis (**Figure 10**). These biological behaviors are regulated by the PPAR, TNF, apoptosis, necroptosis, prolactin, and MAPK signaling pathway, among others. The molecular docking studies and the dynamic simulations verified the potential of CoQ10 as a promising anti-aging agent on oocytes.

## Data Availability Statement

The original contributions presented in the study are included in the article/**Supplementary Material**. Further inquiries can be directed to the corresponding authors.

## Author Contributions

QZ and YZ conceptualized the manuscript. LY, HW, SS, HX, YC, and ST collected the literature, wrote the manuscript, and made the figures. QZ edited and made significant revisions to the manuscript. All authors contributed to the article and approved the submitted version.

## Funding

This work was financially supported through grants from the National Natural Science Foundation of China (82004003), the Project of Zhejiang Province Scientific Research Foundation (2020ZA078), Science and Technology Projects of Zhejiang Province (2019C03086), the Project of Zhejiang Province Scientific Research Foundation (2022ZQ061), the Medical and Health Science and Technology Plan of Zhejiang Province (2021KY920), and Zhejiang Zhangqin Famous Traditional Chinese Medicine Expert Inheritance Studio Project (GZS2017014).

## Conflict of Interest

The authors declare that the research was conducted in the absence of any commercial or financial relationships that could be construed as a potential conflict of interest.

## Publisher’s Note

All claims expressed in this article are solely those of the authors and do not necessarily represent those of their affiliated organizations, or those of the publisher, the editors and the reviewers. Any product that may be evaluated in this article, or claim that may be made by its manufacturer, is not guaranteed or endorsed by the publisher.

## Abbreviations

BP, Biological process; CAT, Catalase; CC, Closeness centrality; CoQ10, Coenzyme Q10; COC, Cumulus cell-oocyte; DC, Degree centrality; DEG, Differentially expressed gene; EC, Eigenvector centrality; ETC, Electron transport chain; FGSC, Female germline stem cell; GC, Granulosa cell; GO, Gene ontology; GRB2, Growth factor receptor-bound protein 2; GSR, Glutathione reductase, mitochondrial; HMOX1, Heme oxygenase 1; KEGG, Kyoto Encyclopedia of Genes and Genomes; LAC, Local average connectivity-based method; MAPK14, Mitogen-activated protein kinase 14; MD, Molecular dynamic; mRNA, Messenger RNA; mtDNA, Mitochondrial DNA; NC, Network centrality; OSC, Ovarian germline stem cell; OXPHOS, Oxidative phosphorylation; POR, Poor ovarian response; PPARA, Peroxisome; PPI, Protein–protein interaction; Rg, Radius of gyrate; RMSD, Root mean square deviation; ROS, Reactive oxygen species; SASA, Solvent-accessible and surface area; ScRNA-seq, Single-cell RNA sequencing; SMILES, Simplified molecular-input line-entry specification; SQSTM1, Sequestosome-1.
